# The Importance of Autophagy Regulation in Breast Cancer Development and Treatment

**DOI:** 10.1155/2014/710345

**Published:** 2014-09-17

**Authors:** Joanna Magdalena Zarzynska

**Affiliations:** Department of Food Hygiene and Public Health, Faculty of Veterinary Medicine, WULS-SGGW, Nowoursynowska 159, 02-776 Warsaw, Poland

## Abstract

Breast cancer (BC) is a potentially life-threatening malignant tumor that still causes high mortality among women. One of the mechanisms through which cancer development could be controlled is autophagy. This process exerts different effects during the stages of cancer initiation and progression due to the occurring superimposition of signaling pathways of autophagy and carcinogenesis. Chronic inhibition of autophagy or autophagy deficiency promotes cancer, due to instability of the genome and defective cell growth and as a result of cell stress. However, increased induction of autophagy can become a mechanism which allows tumor cells to survive the conditions of hypoxia, acidosis, or chemotherapy. Therefore, in the development of cancer, autophagy is regarded as a double-edged sword. Determination of the molecular mechanisms underlying autophagy regulation and its role in tumorigenesis is an essential component of modern anticancer strategies. Results of scientific studies show that inhibition of autophagy may enhance the effectiveness of currently used anticancer drugs and other therapies (like radiotherapy). However, in some cases, the promotion of autophagy can induce death and, hence, elimination of the cancer cells and reduction of tumor size. This review summarizes the current knowledge on autophagy regulation in BC and up-to-date anticancer strategies correlated with autophagy.

## 1. Introduction

Breast cancer (BC) is the most common and fatal cancer in women worldwide, despite decreasing mortality rates that result mostly from efficient screening strategies [1, 2]. It has been estimated that approximately 1.3 million females develop BC each year, with around 465.000 expected to succumb to the disease [3, 4]. BC is ranked in the second place in mortality among cancer types [5], causing death of about 350,000 women in both developed and developing countries every year [6]. More than 90% of lethality in patients is caused by metastasis, and the occurrence of distant metastases (distinct metastatic pattern involving the regional lymph nodes, bone marrow, lung, and liver) severely limits the prognosis [7–9]. The 5-year survival rate for patients with BC drops sharply from 98% for individuals with localized disease to 23% for those with metastatic disease [10]. Many factors are involved in the pathogenesis and progression of BC, including genetic, biological, and environmental factors, as well as lifestyle [6]. It has been estimated that 75% of women with sporadic invasive BC have no known epidemiological risk factors [11]. On the other hand,* BCL-2* protooncogene is overexpressed in half of all human malignancies and more than 60% of BC and is thought to exert its oncogenic role by preventing cells from undergoing apoptosis [12].

Recent studies demonstrated association between autophagy and cancer. Autophagy is a genetically regulated process, controlled by a group of evolutionarily conserved genes called* ATG* (autophagy-related genes). At least 11* ATG* genes identified in yeast have orthologs in mammals. Autophagy initially was identified as a cell survival mechanism to protect cells from nutrient deprivation. It maintains proteins and organelles turnover and by that ensures homeostasis. Removing excess or damaged intracellular components in response to stress, as well as microbes, allows cells to restrain damage, including alternations in the genome (genome instability), and subsequent inflammation. Suppressing of genome instability limits initiation and progression of cancer. In certain developmental conditions, in cell's response to metabolic stress, but also under cytotoxic stimuli, autophagy results in a form of cell death described as type II programmed cell death [13]. This could be seen also, for example, in cells expressing BCL-2 or Bcl-xl or lacking both Bax and Bak proteins.

The role of autophagy in tumorigenesis and treatment responsiveness is complicated and context-dependent and presumably differs in different stages of cancer development. At the initial stages of cancer development, autophagy may represent a protective response thanks to its catabolic roles, by degrading and/or recycling cell components, like damaged organelles and misfolded proteins [14–16]. Autophagy may hinder proliferation of cells with cancer-linked mutations. It can also limit propagation of this type of mutations, thus suppressing tumorigenesis by facilitating senescence (biological aging). However, once a tumor develops, the cancer cells can utilize autophagy for their own cytoprotection. Autophagy can increase oxidative stress, thereby promoting genome instability and malignant transformation [15–18], and cancer cells can use enhanced autophagy to survive under metabolic and therapeutic stress [16]. Additionally, it has been suggested that metastatic cancer cells may escape from anoikis (process of apoptosis induced by lack of correct cell-extracellular matrix attachment) via the induction of autophagy [19, 20].

Current BC therapy depends on the type and stage of the BC and traditionally consists of a multivariate approach including surgery, hormone therapy, systemic chemotherapy, radiotherapy, and molecular targeted therapy [2, 9]. Despite treatment, the majority of breast cancers are incurable and ultimately claim the life of the patient with complications and development of chemoresistance [9]. The pharmacological or genetic inhibition of autophagy is studied and correlated with sensitization of cancer cells to the lethal effects of various cancer therapies including chemotherapy, radiotherapy, and targeted therapies, suggesting that suppression of the autophagic pathway could represent a valuable strategy for cancer treatment. Continuing progress in this field will be critical for developing new cancer therapies and improving those already in use [21, 22].

## 2. Autophagy Outline

Currently, over 35 proteins are believed to be essential for autophagy occurrence and progression [23]. Biological and morphological changes have been observed under the influence of autophagic pathway [24]. Under stress conditions autophagy is induced to keep the balance in cells [25]. Cellular stress can be caused by a variety of chemical and physical agents, like nutrient starvation, proinflammatory state, hypoxia, oxidants, infectious agents, and xenobiotics [26, 27].

The complete macroautophagic (referred to hereafter as autophagy) flow is generally divided into the following stages: induction, vesicle nucleation, vesicle elongation and completion, docking and fusion, degradation, and recycling [26, 28, 29]. Basal levels of macroautophagy are kept in check by mTORC1 (mammalian target of rapamycin complex 1) phosphorylation of* ATG13* (autophagy-related gene 13) and ULK1 (uncoordinated 51-like kinase 1/*ATG1*) or ULK2. It inhibits ULK1 phosphorylation of FIP200 (focal adhesion kinase interacting protein of 200 kD/*ATG17*) [30, 31]. The mTORC1 complex is an important component of a network that senses the nutrient state of the cell and accordingly maintains homeostasis by controlling the levels of anabolism and catabolism. High levels of amino acids maintain mTORC1 in an active state by enhancing its binding to regulatory proteins Rag and Rheb (Ras homolog enriched in brain) GTPases (guanosine triphosphatases) [32]. Insulin and IGF1 (insulin-like growth factor 1) indirectly induce mTORC1 activity by stimulating class 1 PI3K (phosphoinositol 3-kinase) production of PIP3 (PtdIns(3,4,5)P3), which induces Akt kinase (protein kinase B) at the plasma membrane, which in turn activates mTORC1 by inhibiting TSC (tuberous sclerosis complex) proteins 1/2, thereby relieving their repression of Rheb [28]. Low glucose levels or high levels of AMP (adenosine 5′-monophosphate), which indicate low cellular energy status or stress, activate AMPK (AMP-activated protein kinase), which inhibits mTORC1 and stimulates macroautophagy [26, 27, 33, 34].

Vesicle nucleation is the initial step in which proteins and lipids are recruited for construction of the autophagosomal membrane. In mammalian cells, this process is initiated by activation of the class III PI3K/Beclin-1 complex, including the core members hVps34/PIK3C3, Beclin-1, and p150. Numerous additional binding partners of this complex function as either positive or negative regulators and include BAX-interacting factor-1 (BIF-1), ATG14L, UVRAG (UV irradiation resistance-associated gene), Ambra1 (activating molecule in Beclin-1-regulated autophagy protein 1), and Rubicon [23, 26, 29, 35, 36]. Additionally, Ambra1 is required for the interaction of Beclin-1 with PI3KC3/Vps34 [36]. Interaction of UVRAG with Beclin-1 leads to increased PI3KC3/Vps34 kinase activity, which results in increased autophagic initiation. UVRAG is also involved in the interaction of Bif with Beclin-1, which facilitates the activation of PI3KC3/Vps34. Rubicon (RUN domain Beclin-1-interacting cysteine-rich-containing protein) has also been shown to negatively regulate autophagy [37, 38]. The regulation of the maturation process of the autophagosome is multifactorial and involves Rab GTPase, SNAREs (soluble N-ethylmaleimide-sensitive fusion attachment protein receptors), and ESCRT (endosomal sorting complexes required for transport) proteins, molecules of the acidic lysosomal compartment (e.g., v-ATPase, LAMP proteins-lysosome-associated membrane glycoproteins, lysosomal carriers, and hydrolases), and Beclin-1 [39].

## 3. Autophagy Regulation in Breast Tumors

Autophagy seems to have different roles in cancer cells. Autophagy plays a complex role in tumor initiation and progression. It protects against the deleterious effects of reactive oxygen species in the cells, which may lead to mutations in DNA, and promotes cell transformation [40]. Autophagy has been shown to be required for the transformation of mouse embryonic fibroblasts by the Ras oncogene and this effect is linked to its role in nutrients recycling, such as glucose uptake and increased glycolytic flux [41, 42]. During the later stages of* in vivo* tumor formation, autophagy is necessary for cancer cells survival in hypoxia conditions before the vascularization of tumor takes place [40, 42]. In fully transformed cancer cells it appears to function as a tumor suppressor, as defective autophagy is associated with malignant transformation and carcinogenesis. However, in normal cells and in some cancer cells the induction of autophagy is associated with cell death [12]. Studies have shown that cancer cells express lower levels of the autophagy-related proteins LC3-II and Beclin-1 than normal epithelial cells and that while heterozygous disruption of* BECN1* promotes tumorigenesis, the overexpression inhibits tumorigenesis, supporting the assertion that defective autophagy or inhibition of autophagy plays a role in malignant transformation [12].

Regulation of autophagy in tumors is governed by principles similar to normal cells, only in a much more complicated manner, given the frequently observed abnormal PI3K activation in cancer and the multitude of interactions between the PI3K/Akt/mTOR pathway and other cell signaling cascades, often also deregulated in tumor cells [35]. A deregulated PI3K/Akt/mTOR axis not only suppresses autophagy but also induces protein translation, cell growth, and proliferation, a major driving force in tumorigenesis. Tumors with high metabolic demands, with constitutively active PI3K mutations, PTEN loss, or Akt activation, would be expected to be dependent on autophagy for energy homeostasis and survival. Suppression of autophagy by the PI3K signaling cascade presents a disadvantage for rapidly proliferating tumor cells and leads to the prediction that compensatory mechanisms (like deregulated apoptosis and/or metabolism) might be concurrently activated to prevent the negative implications of defective autophagy on tumor cell survival. Ras/Raf/ERK pathway is among the most commonly deregulated pathways identified in tumors, as indicated by frequently observed activating mutations in Ras or B-Raf oncogenes [13].

Many proteins and active factors correlated with autophagy are reported to be associated with human cancers [43]. Autophagic cell death has been described, for example, in antiestrogen-treated cultured human mammary carcinoma MCF-7 cells [44]. The role of autophagy might be different in certain stages and aspects of tumor development. Various tumor suppressors (e.g., PTEN, TSC1/2, p53, and DAPK) are autophagy inducers, whereas some inhibitors of autophagy (e.g., Akt and Ras) possess oncogenic activity [45]. Studies of Kadota et al. [46] and Kim et al. [47] showed that the more advanced stages of breast cancer overexpress several other oncogenic and signaling proteins, such as IGF-1R, Cyclin D1, c-myc, pERK, Stat3, and Pak4, some of which are known activators of Akt-mTOR pathway. ERK activity has also been associated with autophagy and autophagic cell death in many cellular models in response to different stresses [13] and also in TNF-*α* (tumor necrosis factor-alpha) treatment in MCF-7 cells. Several other autophagy regulators in cancer cells, like mitogen-activated kinases (BNIP3) [48] and HSpin 1 (human homologue of the* Drosophila* spin gene product) [49], play a critical role.

PTEN has a stimulatory effect on autophagy, by downregulating PI3K/Akt signaling. It is a critical regulator of the PI3K pathway [39], which selectively hydrolyzes PIP3 to PIP2, and inhibits the activation of Akt/PKB. Akt inhibition leads to suppression of mTOR signaling and the induction of autophagy [39]. Unlike the Ras/Raf/Erk and PI3K pathways, AMPK (AMP-activated protein kinase) pathway has a negative effect on mTOR signaling and promotes autophagy. Upon starvation and activation of calcium signaling, AMPK phosphorylates and activates TSC2, which inhibits mTOR signaling.

EI24/PIG8 (etoposide induced gene) can be mentioned as a critical factor of autophagic degradation, which remains under control of p53 [50, 51]. p53 is well known as a critical tumor suppressor, which protects organism by initiation of cell-cycle arrest, removal of cells with incurred DNA damage, senescence, and apoptosis.* TP53* is the most commonly deleted or mutated gene in human cancers [52]. p53 target genes, effectors of p53 apoptotic response, are currently widely studied. The human EI24 genomic locus is on chromosome 11, in region frequently altered in cancers, and was reported to be mutated in aggressive breast cancers. Furthermore, since EI24/PIG8 (induced by p53) is also known as important apoptotic effector [50, 52], this role may contribute to tumor suppression. Loss of EI24/PIG8 was associated with tumor invasiveness but not with the development of the primary tumor [52].

### 3.1. Beclin-1

The most important evidence linking dysfunctional autophagy and cancer comes from studies demonstrating that the inhibition of autophagy in mice, by disruption of* BECN1*, increases cellular proliferation, increases the frequency of spontaneous malignancies (i.e., lung cancer, liver cancer, and lymphomas) as well as mammary hyperplasia, and accelerates the development of carcinogen-induced premalignant lesions [12].

FISH analysis of human breast cancer cell lines using the Beclin-1-containing PAC 452O8 as a probe revealed that 9 out of 22 cell lines had allelic Beclin-1 deletions [35]. Ectopic expression of Beclin-1 restores full autophagy potential in MCF-7 cells, which are tetraploid but have three Beclin-1 copies, and slows cell proliferation* in vitro* and in xenograft tumors* in vivo*. Monoallelic deletion of* BECN1* has been detected in 40–75% cases of human breast, ovarian, and prostate tumors [15, 27, 53], and thus Beclin-1 is considered as a tumor suppressor gene [54]. The aberrant expression of Beclin-1 in many kinds of tumor tissues correlates with poor prognosis [15]. Accordingly, heterozygous deletion of* BECN1* in mice (*BECN1+/−*) resulted in increased incidence of spontaneous tumors [54]. Many breast carcinoma cell lines, although polyploidal for chromosome 17 (*BECN1* gene is placed in 17q21 loci), exhibit deletions of one or more* BECN1* alleles, and human breast tumors show decreased Beclin-1 levels compared to normal adjacent tissue. Restoration of Beclin-1 and autophagy in MCF-7 cells is associated with inhibition of MCF-7-induced tumorigenesis in nude mice [54].* BECN1+/−* mice do not have increased incidence of mammary tumors but rather are susceptible to lymphomas and carcinomas of the lung and liver after long latency. Tumors forming in* BECN1+/−* mice express wild-type* BECN1* mRNA and protein, indicating that Beclin-1 is a haploinsufficient tumor suppressor [35, 36, 53]. Furthermore, Beclin-1 and EI24 alter expression of several autophagy proteins, such as ATG5 and UVRAG [15].

One of the latest factors correlating autophagy and BC is adipokines, autocrine, endocrine, and paracrine-acting bioactive molecules [55]. The adipokine secreted in greatest abundance is adiponectin (AdipoQ). The prevalent evidence indicates that low levels of AdipoQ in the circulation indicate poorer BC risk and prognosis. AdipoQ in breast tissue has a direct anticarcinogenic effect at the site of tumor growth. AdipoQ is potentially able to regulate autophagy through AMPK, whose activation has been observed in breast cancer cells [55, 56]. Liu and colleagues [57] observed that AdipoQ caused upregulation of autophagy in MDA-MB-231 cells* in vitro* and* in vivo*.

### 3.2. MicroRNA

MicroRNAs (miRNAs) are small RNA molecules. Unregulated miRNAs of lymphoma, prostate, lung, and breast cancer have been also detected in blood plasma and serum; circulating miRNAs are currently assessed as proxy biomarkers for BC [58]. There are many pieces of evidence that miRNAs can influence autophagy process in BC cells in many points. MiR-20a, miR-101, miR-106a/b, and miR-885-3p have been reported to have direct possibility to target ULK1/2 [59]. Conserved and predicted binding sites for miR-885-3p exist in* MATG13*,* ATG9A*, and* ATG2B* [29]. MiR-155 might target multiple players in mTOR signaling, including Rheb, RICTOR (RPTOR independent companion of mTOR), and RPS6KB2 (ribosomal protein S6 kinase). MiR-30a and miR-519a can directly target Beclin-1 in the autophagic flow, causing its negative regulation, thereby resulting in decreased autophagic activity. This negative regulation of Beclin-1 expression by miR-30a was shown in the* in vitro* study by Zhu et al. [60] on human breast cancer cell lines MDA-MB-468 and MCF-7. Treatment of tumor cells with the mimic of miR-30a decreased the expression of Beclin-1 mRNA and protein, whereas administration of the miR-30a antagomir increased Beclin-1 levels. Furthermore, high expression of miR-30a blunted the activation of autophagy induced by rapamycin [60]. MiR-376b also regulates Beclin-1, and it can also directly target* ATG4C* [29, 61, 62] in MCF-7 cells, because the antagomir-mediated inactivation of the endogenous miR-376b results in an increased level of ATG4C and Beclin-1 [26, 61]. The direct regulation of UVRAG is modulated by miR-374a and miR-630 [63]. The tumor suppressive miR-101 could act as a potent inhibitor of basal, etoposide-induced, and rapamycin-induced autophagy in MCF-7 cells. Frankel et al. [64] used 4-hydroxytamoxifen (4-OHT) to induce cell death in breast-cancer-derived MCF-7 cells (a stimulus to which they are usually resistant), synergistically with miR-101 as an autophagy inhibitor. The miR-101-mediated inhibition of autophagy sensitized breast cancer cells to 4-hydroxytamoxifen-induced apoptotic cell death, and thus miR-101 was suggested to modulate the chemosensitivity of cancer cells [29]. Three components, including STMN1 (stathmin1), RAB5A (Ras-related protein 5A), and ATG4D, have been identified as targets of miR-101, among which the overexpression of STMN1 could partially rescue cells from miR-101-mediated inhibition of autophagy, indicating a functional importance for this target. RAB5A and STMN1 had previously uncertain roles in autophagy. RAB5A has been shown to regulate ATG5–ATG12 conjugation in the autophagosome completion, while STMN1 destabilizes microtubules and plays an important role in cell-cycle regulation [65]. Most likely, at least in breast cancer cells, elevated levels of autophagy, due to the progressive loss of miR-101, have the potential to trigger cancer cell survival [29, 64]. MiR-221/222 might inhibit the cell-cycle inhibitor, p27Kip1, a downstream modulator of PI3KCI/Akt, leading to autophagic cell death in HER2/neu-positive primary human breast carcinoma MCF-7 cells, whereas the ectopic expression of miR-221/222 renders the parental MCF-7 cells resistant to tamoxifen [66, 67].

## 4. Anticancer Therapies Correlated with Autophagy

### 4.1. Cytoprotective and Nonprotective Forms of Autophagy

A number of antineoplastic therapies, including radiation therapy, chemotherapy (e.g., doxorubicin, temozolomide, and etoposide), histone deacetylase inhibitors, arsenic trioxide, TNF*α*, IFN*γ*, imatinib, rapamycin, and antiestrogen hormonal therapy (e.g., tamoxifen), have been shown to induce autophagy as a protective and prosurvival mechanism in human cancer cell lines [12, 68, 69]. In fact, the therapeutic efficacy of these agents can be increased if autophagy is inhibited [12, 70]. The evidence suggests that autophagy leads to cell death in response to several compounds, including rottlerin, cytosine arabinoside, etoposide, and staurosporine, as well as growth-factor deprivation. A link between autophagy and related autophagic cell death has been demonstrated using pharmacological inhibitors (e.g., 3-MA (3-methyl adenine), CQ (chloroquine), bafilomycin A1, or ammonium chloride) and genetic silencing or knockdown (silencing of* ATG5*,* ATG7*,* ATG12*, and* BECN1*) approaches for suppression of autophagy. For example, the knockdown of* ATG5* or* BECN1* in cancer cells containing defects in apoptosis leads to a marked reduction in autophagic cell death (as well as autophagic response) in response to cell death stimuli with no signs of apoptosis [12, 71]. This is connected with* cytoprotective form of autophagy* [72].

Autophagy has also been shown to protect against cellular stress induced by the chemotherapeutic drugs used in cancer treatment (*nonprotective autophagy*) [72]. Furthermore, because autophagy is frequently upregulated in tumors in response to therapy, it may protect the tumors against therapy-induced apoptosis [73]. Huang et al. studied the effect of* PTTG1* inhibition on tumor growth and metastasis.* PTTG1*/securin is an oncogene that is highly expressed in various tumors and has been correlated with tumor invasiveness and poor prognosis. Huang et al. [68] reported that inhibition of* PTTG1* suppressed tumor growth and metastasis* in vitro* and* in vivo*. The group also investigated autophagy induced during radiation in human breast cancer cells expressing different levels of PTTG1 by measuring the expression of MAP1LC3-I and MAP1LC3-II [68]. The results revealed that radiation increased the ratio of MAP1LC3-II/MAP1LC3-I in MDA-MB-231-2A cells (*PTTG1*-knockdown MDA-MB-231 cells) and MCF-7 cells (low* PTTG1* expression), but not in the parental MDA-MB-231 cells, suggesting that radiation induced autophagy in* PTTG1*-depleted cancer cells. These data suggest that autophagy promotes cell survival and plays a decisive role in choosing between radiation-induced senescence and apoptosis. Inhibition of autophagy by 3-MA resulted in the MDA-MB-231-2A cells undergoing apoptosis instead of radiation-induced senescence; cells undergoing apoptosis could have enhanced radiosensitivity. It appears that apoptosis is a more efficient way to trigger cell death, as inhibition of autophagy and senescence by treatment with 3-MA and bafilomycin A1 enhanced cell death [68].

Gewirtz [72] reported that ionizing radiation could promote autophagy in BC cells in cell culture, but autophagy inhibition did not alter sensitivity to radiation. Furthermore, they showed that chloroquine did not sensitize (4T1) murine breast tumor cells to radiation in an immunocompetent animal model. Based on the results obtained it was impossible to determine whether radiation promoted autophagy or the chloroquine actually effectively inhibited autophagy in the tumor-bearing animals. Supposedly, the lack of sensitization could be related to the findings [23] that autophagy inhibition interferes with the immune system's capability to recognize the tumor undergoing a stress response.

Such disclosures have led to several clinical trials involving the use of inhibitors of the autophagy flux (“autophagic flux” represents the synthesis of autophagosomes, transportation of different substrates, and degradation of autophagy inside the lysosome) as a combination therapy [74], to improve the efficacy of radiotherapy in BC patients. For example, hydroxychloroquine (HCQ), an autophagy inhibitor that is currently in phase I and phase II clinical trials, has been used in combination with several chemo- and radiotherapies [28, 68, 75]. HCQ is a less toxic version of CQ and the best autophagy inhibitor currently commercially available for clinical trials [76]. Currently, there are 52 clinical trials of HCQ listed on the United States Government website (clinicaltrials.gov), of which 32 are cancer studies. There are 48 results for HCQ in cancer therapy (2 for breast cancer) of HCQ in combination with a range of chemotherapeutic agents.

Irradiated cancer cells can induce damage in neighboring unirradiated cells by intracellular gap-junction communication or signals that are released outside of the cells [77]. Huang et al. [68] have indicated that radiation-induced senescent MDA-MB-231-2A cells secrete multiple cytokines and chemokines, including CSF2 (colony stimulating factor, expressed in the highest level), CXCL1 (C-X-C motif ligand 1), IL6 (interleukin 6), and IL8 (interleukin 8). These factors are involved in multiple functions during cancer progression. Autophagy inhibition in MDAMB-231-2A cells significantly decreased the release of CSF2, suggesting that autophagy plays an important role in promoting the secretion of SASPs (senescence-associated secretory phenotypes). In support of this notion, it has been reported that inhibition of autophagy delays the secretion of several senescence-associated cytokines, such as IL6 and IL8 [78].

### 4.2. Cytotoxic and Cytostatic Autophagy

The next form of autophagy, which should be taken under consideration in the field of cancer treatment, is* cytotoxic autophagy*. For example, Bristol et al. [79] have reported that vitamin D (or the vitamin D analog, EB 1089) can be combined with radiation to promote a cytotoxic form of autophagy in breast tumor cells (MCF-7 and ZR-75). Other research groups also showed that the generation of* cytotoxic autophagy* may either lead independently to cells death or act as a precursor to apoptosis [80]. Functionally,* cytotoxic autophagy* is associated with a reduction in the number of viable cells and/or reduced clonogenic survival upon treatment [81]. Gewirtz [81] identified an additional form of autophagy, termed* cytostatic autophagy*, in nonsmall cell lung cancer cells (A549 and H460), which was induced in similar conditions to the ones previously described in regard to breast tumor cells. Similarly to the impact on* cytotoxic autophagy* in breast tumor cells, pharmacologic inhibition of autophagy with either chloroquine or 3-MA protected the cells from the sensitization to radiation by vitamin D or EB 1089. What distinguishes cytostatic autophagy from the cytoprotective form is the failure to detect evidence of cell killing reported in the breast tumor cells [81]. The group of both Gewirtz [81] and Kroemer had demonstrated cytoprotective autophagy by radiation alone [82], but the addition of vitamin D or EB 1089 converted cytoprotective autophagy to cytostatic autophagy.

As Ras/Raf/ERK pathway belongs to the most commonly deregulated pathways identified in tumors, it is currently the target of new antitumor strategies, based on the inhibition of upstream ERK regulators. However, because ERK activation is implicated in DNA-damaging agent-induced cell death, inhibiting ERK activity in combination therapy with classical antitumor compounds might affect the efficiency of such compounds. For example, in MCF-7 human breast adenocarcinoma cell line such combined therapies with doxorubicin [83], tamoxifen [84], taxol [85], or Δ Raf1 [86] and TNF*α* [87] were used. Targeting autophagy was used to circumvent TRAIL-resistance in tumors with apoptosis defects; knockdown of autophagy, in combination with tamoxifen or 4-hydroxy-tamoxifen (4-OH-T), resulted in decreased cell viability of two human hormone-dependent breast cancer cell lines, ER-positive MCF-7 [70, 88] and T-47D cells [89]. Tamoxifen, the most commonly used antiestrogen, exerts its pharmacological action by binding to estrogen receptor alpha (ER*α*) and blocking the growth promoting action of the estrogen bound receptor in BC cells. However, the development of antiestrogen resistance has become a major impediment in the treatment of ER-positive BC. Samaddar et al. [89] had reported that autophagy plays a critical role in the development of antiestrogen resistance, and overexpression of Beclin-1 downregulated estrogenic signaling and growth response [90].

### 4.3. Silencing Autophagic Genes

Genetic approaches could be represented by some studies using gene silencing to receive therapeutic effect via cell death induction. For example, the* BCL-2* protooncogene is overexpressed in half of all human malignancies and more than 60% of BC. It exerts its oncogenic role by preventing cells from undergoing apoptosis.* BCL-2* overexpression not only leads to the resistance of cancer cells towards chemotherapy, radiation, and hormone therapy but also causes an aggressive tumor phenotype in patients with a variety of cancers [12]. Recent findings suggested that silencing* BCL-2* expression by siRNA in MCF-7 cells led to significant autophagic, not apoptotic, cell death [39, 71]. It has been demonstrated that the knockdown of autophagy genes (e.g.,* ATG5* and* BCN1*) significantly inhibited both autophagy and cell death induced by* BCL-2* siRNA after a long-term treatment of up to seven days [71]. MCF-7 cells are known to be caspase 3-deficient, providing a higher threshold for the induction of apoptosis, potentially rendering the autophagic cell death pathway more important. Furthermore, about 45–75% of tumor tissues from BC patients do not have detectable caspase 3 expression [91]. Additionally, Akar et al. [71] reported that doxorubicin predominantly induced autophagy at low doses and apoptosis at high doses. Furthermore, the combination of* BCL-2* siRNA treatment with a low dose of doxorubicin enhanced the autophagic response, tumor growth inhibition, and cell death. It was the first evidence that targeted silencing of* BCL-2* induces autophagic cell death in BC cells, which constitutes a good beginning for further research on this type of alternative therapeutic strategies.

### 4.4. Pharmacological Approach to the Antitumor Autophagic Therapies

Studies on the role of autophagy in tumor development and progression have led to a subsequent progress in pharmacological approach to the antitumor autophagic therapies, which aim to activate or inhibit autophagy. Many drugs and compounds that modulate autophagy are currently receiving considerable attention [15, 43]. These include, for example, autophagy inducers such as mTORC1 inhibitor rapamycin [15] and its analogs that are called rapalogs, such as Everolimus (RAD001), which are also often used as tools to study autophagy process [28, 74]. Everolimus was shown to enhance the sensitivity of tumors to radiation by induction of autophagy [28].

Also natural products are considered as potential anticancer candidates, being direct or indirect sources of new chemotherapy adjuvants to enhance the efficacy of chemotherapy and/or to ameliorate its side effects [92]. Lu et al. [92] have used Cyclovirobuxine D (CVB-D), an alkaloid component isolated from the roots of* Buxus microphylla* var.* sinica* (recently used for the cardiovascular diseases treatment) as a potential inducer of autophagy in MCF-7 cell line. After exposure of the breast cancer cells to CVB-D (10 mM) for 24 h, an induction of cell death was noted in a concentration- and time-dependent manner. It was accompanied with parallel increase in the level of autophagy with accumulation of autophagosomes (upregulated LC3II). Then to explore whether autophagy was involved in the cytotoxicity of CVB-D, 3-MA was used. The results of Western blotting and MDC staining showed that autophagy promoted by CVB-D was significantly attenuated by 3-MA [92].

Another team, Lu et al. [93], was working on the sesquiterpene lactone, PTL (bioactive component in feverfew, used for fever, migraine, and arthritis). They have reported that PTL increased ROS level, activated AMPK, and induced autophagy in MCF-7 cells and that inhibition of AMPK or autophagy significantly potentiated PTL-induced apoptosis in MCF-7 breast cancer cells. Moreover, PTL showed similar effects on other BC cell lines, MDA-MB-231 and T47D, and again usage of 3-MA suppressed PTL-induced autophagy.

The more challenging issue is the monitoring of autophagic activity in humans, in tissue and blood samples. It seems to be more important to measure autophagic flux than autophagosome number. However, till now, measurement of autophagic flux in paraffin-embedded tissue samples has been unsuccessful, and even the detection of endogenous LC3-II (commonly used marker for autophagosomes) is problematic in tissue sections [15].

## 5. Concluding Remarks

There has been a tremendous amount of progress in our understanding of the role of autophagy in cancer. Overall, the data support a dynamic role of autophagy in cancer, both as a tumor suppressor early in progression and later as a protumorigenic process, critical for tumor maintenance and therapeutic resistance. The specification of the autophagic cargo in tumors with increased autophagy is important for understanding the changes in metabolism between normal and malignant cells. Undoubtedly, progress in genomics, proteomics, and metabolomics will be helpful in this scope. Currently, the molecular mechanisms underlying the regulation of autophagy and the role of autophagy in cancer cells are not fully understood but are progressively revealed. Defects in apoptosis lead to increased resistance to chemotherapy, radiotherapy, and other therapies. Therefore, induction of autophagic cell death may be an ideal approach in these resistant cancers therapies. Continued progress in this field will be critical in developing new cancer therapies and improving those already in use.

As was presented in this review, most experiments regarding BC are carried out on cell lines,* in vitro*. Autophagy inhibition by HCQ in combination with chemotherapy is currently being evaluated in multiple ongoing clinical trials in patients with solid tumors, but we should take into account that autophagic effect is context-dependent. While tumor cell susceptibility to autophagy may depend on tumor genotype and the therapeutic agents utilized, data are very limited and it remains unclear whether such new strategies will be clinically beneficial.
